# The clinical value of ERCP-guided cholangiopancreatoscopy using a single-operator system

**DOI:** 10.1186/s12876-019-0953-9

**Published:** 2019-02-26

**Authors:** Marcus Reuterwall, Jeanne Lubbe, Lars Enochsson, Lars Lundell, Magnus Konradsson, Frederik Swahn, Marco Del Chiaro, Matthias Löhr, Urban Arnelo

**Affiliations:** 1 Division of Surgery, CLINTEC, Karolinska Institutet, Center for Digestive Diseases, Karolinska University Hospital, Huddinge, 114 86 Stockholm, Sweden; 2 Department of Surgery, Skåne University Hospital, Lund, Sweden; 3 Division of Surgery, University of Stellenbosch, Tygerberg Hospital, Cape Town, South Africa

**Keywords:** Per-oral, Single-operator, Cholangiopancreatoscopy, Clinical value, ERCP, Spyglass, Bile ducts, Pancreatic ducts

## Abstract

**Background:**

Single-operator, per-oral cholangiopancreatoscopy (SOPCP) enables direct biliopancreatic ductal visualization, targeted tissue sampling, and therapeutic intervention. At Karolinska University Hospital, SOPCP was introduced early and has since been extensively utilized according to a standardized protocol. We analysed the clinical value of SOPCP in the diagnosis and treatment of biliopancreatic diseases in a single high volume center.

**Methods:**

All SOPCP procedures performed between March 2007 and December 2014 were retrospectively reviewed. Each procedure’s diagnostic yield and therapeutic value was evaluated using a predefined 4 grade scale; 1 - no diagnostic or therapeutic value, 2 - information gained did not impact clinical decision-making and in case of a therapeutic intervention, did not alter the clinical course of the patient, 3 - information gained had an impact on clinical decision-making and in the case of a therapeutic intervention, assisted subsequent disease management, and finally, 4 - information gained was essential and critical for clinical decision-making and in case of a therapeutic intervention, solved the clinical problem requiring no further therapeutic actions. Descriptive statistics were used to analyse results, with uni- and multivariate analyses completed to assess risk of adverse events.

**Results:**

During the study period, 365 SOPCP procedures were performed. We found SOPCP of pivotal importance (grade 4) in 19% of cases, and of great clinical significance (grade 3) in 44% of cases. SOPCP did not affect clinical decision-making or alter clinical course (grade 1 and 2) in 37% of cases.

**Conclusion:**

SOPCP offers direct access to the biliopancreatic ducts for both diagnostic and therapeutic purposes, adding significant clinical value in 64% of cases.

**Trial registration:**

As this is a purely observational and retrospectively registered study in which the assignment of the medical intervention was not at the discretion of the investigator, it has not been registered in a registry.

## Background

Technological advancement in recent years has led to per-oral cholangiopancreatoscopy emerging as a solution to the challenge of diagnosis and treatment in the small and relatively inaccessible biliopancreatic ductal systems. As an adjunct to endoscopic retrograde cholangiopancreatography (ERCP), it can be performed in one of three ways [1]; dual-operator or mother-baby cholangioscopy, direct per-oral cholangioscopy, or single-operator (catheter-based) cholangiopancreatoscopy (SOPCP). There is an international trend towards the use of the single-operator system, mainly due to ease of use [2]. The SpyGlass Direct Visualization System (Boston Scientific, USA) makes use of a standard duodenoscope, enabling a single operator to insert specially designed optical fibres via an optical port, with two further available ports, one for irrigation and the other to function as a working channel for insertion of the biopsy forceps, lithotripsy fibres or laser probe [3].

The use of SOPCP has been evaluated both for the diagnosis of indeterminate bile duct strictures, as well as for the treatment of ‘difficult’ bile duct stones [4–9]. It has recently been successfully utilized in patients with primary sclerosing cholangitis (PSC), enabling targeted biopsies and navigation of otherwise inaccessible strictures [10]. Additionally, it offers unique options for visualization, surveillance and early-detection of premalignant lesions in pancreatic ductal epithelium, being particularly relevant for lesions such as intraductal papillary mucinous neoplasms (IPMN) [11–16].

Since its introduction, SOPCP has been met with divergent experiences; on the one hand it is considered to be a valuable diagnostic and therapeutic tool, with the other extreme the view that it might represent a dangerous acquisition of redundant information. Although procedural success when using SOPCP has been established [7, 17], and adverse events described [2], the degree to which SOPCP specifically alters clinical management remains to be investigated. At Karolinska University Hospital, SOPCP has been utilized since 2007, following a standardized protocol. Accordingly, we have collected a substantial longitudinal experience. This report critically analyzes our data, focusing on the clinical utility of this technology in the diagnosis and treatment of biliopancreatic disease.

## Methods

From 2007 to 2014, all patients undergoing SOPCP with the SpyGlass Direct Visualization System at the Karolinska University Hospital, were included for study. Unless the indication for investigation was complex cholelithiasis, all patients were discussed at a multidisciplinary team meeting, where an individualized management plan was decided on. Complex cholelithiasis was defined as either ‘difficult’ to remove common bile duct stones (stone removal not achieved by conventional means), or intrahepatic stones. Baseline demographics included a physical status grading according to the American Society of Anesthesiologists (ASA) classification system where grades indicate the presence of; I – a normal healthy patient, II – mild systemic disease, III – severe systemic disease, and IV – systemic disease a constant threat to life. Each procedure’s diagnostic yield and therapeutic value was retrospectively reviewed using a predefined graded scale as follows; 1 - no diagnostic or therapeutic value, 2 - information gained did not impact clinical decision-making and in case of a therapeutic intervention, did not alter the clinical course of the patient, 3 - information gained had an impact on clinical decision-making and in case of a therapeutic intervention, assisted subsequent disease management, and finally, 4 - information gained was essential and critical for clinical decision-making and in case of a therapeutic intervention, solved the clinical problem requiring no further diagnostic or therapeutic actions. The scale was applied to each individual case by a single independent reviewer, and the final decision as to the assigned grade was made by determining the impact the procedure had on the final multidisciplinary team meeting decision.

All patients routinely received antibiotic prophylaxis consisting of either intravenous piperacillin+tazobactam or oral sulfonamid+trimethoprim, administered prior to the ERCP. According to standard endoscopy suite protocol, prophylactic non-steroidal anti-inflammatories were not administered and in PSC patients brush samples for cytology and flow cytometry [10] were obtained. Detailed criteria for tissue sampling of strictures by mini-forceps were not defined, and SOPCP-guided biopsies were taken at the discretion of the endoscopist whenever suspicious focal findings were present.

All procedures were carried out under general anaesthesia with the SpyGlass Direct Visualization System (Boston Scientific, USA) passed through a standard duodenoscope. The first generation Spyglass System consists of three components; firstly a reusable SpyGlass fibre optic probe (allowing direct visual guidance and examination of the respective duct systems), secondly the SpyScope disposable access and delivery catheter system (capable of accommodating both optical and accessory devices used in the biliary system), and finally the disposable mini-biopsy forceps (used to capture tissue specimens for histomorphologic diagnosis). The fibre optic probe has an outer diameter of 0.9 mm, image transmission of 6000 pixels, a 0^O^ direct view, and a field view of 70^o^. The light source is a Xenon light connected to the SpyScope catheter. Electrohydraulic lithotripsy was performed using either a 1.9-Fr coaxial electrode probe (Olympus Lithotron EL-25, 1000 mJ; Olympus Inc., Stockholm, Sweden) or Nortech AUTOLITH® EHL-generator with 1,9 F bipolar biliary EHL probe (Northgate Technologies Inc. Elgin, IL, USA).

After successful cannulation of the papilla and positioning of the guide wire into the ductal system under investigation, a ductogram was obtained. An endoscopic sphincterotomy was performed in all patients. In case of an insufficient previous sphincterotomy, a redo sphincterotomy was performed. The Spyglass system was then carefully advanced into the relevant duct, with saline irrigation used for clearance of debris.

In patients with indeterminate strictures, SOPCP was applied for visually targeted biopsies in cases where intraductal papillary and nodular structures or irregular mucosal surfaces could be visualized. The presence of dilated tortuous or irregular tumor vessels, if present, was information that could be added to the clinical data in each case, and was taken into account at subsequent multidisciplinary discussion. For the endoscopic diagnosis of IPMN, previously defined criteria were used [13, 15]. When discrete, circumscribed areas containing finger like protrusions, mucus or tumour vessels were identified, biopsy specimens were obtained from several different parts of the lesion under direct vision. In the absence of discrete lesions, random samples from the ductal epithelium were taken and sent for examination by a pathologist. In many of these patients brush cytology specimens were also obtained.

Intra- and postprocedural adverse events were graded according to the American Society for Gastrointestinal Endoscopy (ASGE) severity grading system [18].

Descriptive statistics were used such as frequencies, median values, and ranges. Uni- and multivariate analyses were done to address risk factors for the occurrence of postprocedural adverse events. All analyses were carried out using STATA 13.1 (StataCorp LP, College Station, Texas, USA).

The study protocol was approved by the regional research ethics committee at Karolinska Institutet, Stockholm, Sweden (dn 2014/55–31/4).

## Results

During the 7-year period, 365 Spyglass procedures were completed in 311 patients. Demographic details of patients undergoing SOPCP are represented in Table 1. The majority of patients had only one procedure. The median duration of SOPCP was 99 min (range 50–275 min), including procedures such as ERCP and endoscopic ultrasound (EUS) performed during the same anaesthetic. This suggests a certain degree of complexity and time needed for completion.

**Table 1:** Patient demographics for single-operator, per-oral cholangiopancreatoscopy (SOPCP)

Total SOPCP procedures	365
Number of patients	311
Patients undergoing a single procedure	273 (88%)
Patients undergoing multiple procedures	38 (12%)
Female gender	137 (44%)
Age	64 (4–94)
Referrals from outside Stockholm	103 (33%)
Duration of procedures in minutes	99 (50–275)
ASA 1	58 (16%)
ASA 2	186 (51%)
ASA 3	121 (33%)
ASA 4	0 (0%)

Figures are based on numbers of SOPCP procedures as n (%) or median (minimum-maximum). *ASA* American Society of Anesthesiologists

Specific indications for patients undergoing SOPCP are shown in Table 2. The main indication was indeterminate bile duct strictures in non-PSC patients. Complex cholelithiasis, was an indication for the procedure in only 16% of the cases. In 71% of our patients, the bile duct was the main target, the pancreatic duct in 24%, and both ducts in 5%.

**Table 2:** Indications for single-operator, per-oral cholangiopancreatoscopy (SOPCP)

Complex cholelithiasis	58 (15.9%)
Indeterminate strictures (non-PSC patients):	119 (32.6%)
Indeterminate strictures (PSC patients)	82 (22.5%)
Cystic lesions of the pancreas (including IPMN)	64 (17.5%)
Chronic pancreatitis ± lithotripsy	20 (5.5%)
Miscellaneous	22 (6%)

Figures are based on numbers of SOPCP procedures as n (%)

In 291 (79.6%) patients the procedure could be performed in an outpatient setting. Intra- and post procedural adverse events are presented in Table 3. We found an overall adverse event rate (AER) of 16%, with the majority of these being scored as mild or moderate.

**Table 3:** Adverse events after SOPCP

	N (%)
All adverse events	59 (16.2)
-mild	33 (9.0)
-moderate	22 (6.0)
-severe	3 (0.8)
-fatal outcome	1 (0.3)
Pancreatitis (including one fatal case)	29 (7.9)
-mild	13 (3.6)
-moderate	14 (3.8)
-severe	1 (0.3)
Cholangitis	16 (4.4)
-mild	9 (2.5)
-moderate	7 (1.9)
-severe	0 (0)
Miscellaneous (including one perforation that required surgical repair)	14 (3.8)
-mild	11 (3.0)
-moderate	1 (0.3)
-severe	2 (0.5)

Adverse events classified according to the ASGE guidelines [18]

The most frequent adverse event was pancreatitis in 8% of cases, with an equal distribution between mild and moderate pancreatitis. We were unable to demonstrate a change over time regarding the risk for this complication. Cholangitis was recorded in 16 patients (4%), of which no cases were severe. We experienced one fatal adverse event which was due to severe pancreatitis. In this patient an endoscopic ultrasound-guided puncture of a cystic pancreatic lesion had been performed in combination with the SOPCP. Initially a gastro-intestinal perforation was suspected, but this could not be verified on imaging. The clinical course was complex, ending in multi-organ failure and death on day 101. The subsequent autopsy revealed severe necrotizing pancreatitis.

When analysing specific risk factors for the occurrence of postprocedural adverse events, we found that pancreatoscopy was associated with an AER of 19.8% as compared to 9.6% for cholangioscopy. In the pancreatoscopy group we furthermore found a non-dilated main pancreatic duct in 9 of the 17 pancreatitis cases (53%).

**Fig. 1:**
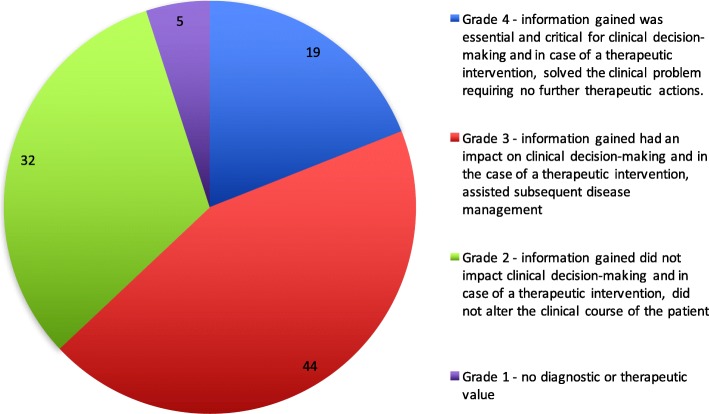
The relative (%) distribution of diagnostic yield and therapeutic value as scored according to the predefined 4 grade scale

When the clinical value of the respective SOPCP procedures were carefully scrutinised, a significant gain (grade 3–4) was detected in 64% of the cases (Fig. 1). The largest number of grade 2 procedures were due to an inability to definitively ascertain the relative contribution of the information provided by the SOPCP, in the presence of multiple factors that ultimately affected the clinical decision making process (*n* = 54).

Table 4 is a representation of the assigned grades (grouped as grade 1–2, or grade 3–4) according to the individual indications for SOPCP. In 79% of procedures performed for the treatment of complex bile duct stones, therapeutic value was graded as 3–4, whereas in 66% of procedures performed as part of work-up for cystic pancreatic lesions, diagnostic yield was graded as 3–4. For the evaluation of indeterminate biliary strictures, both in non-PSC and PSC patients, the diagnostic yield was graded as 3–4 in 57 and 56% of cases respectively. The clinical value of SOPCP in patients with chronic pancreatitis was graded as 1–2 in 55% of patients.

**Table 4:** Representation of assigned grades

Indication	Total n	Grades 1–2 n (%)	Grades 3–4 n (%)
Complex cholelithiasis	58	12 (20.7)	46 (79.3)
Indeterminate strictures (non-PSC patients):	119	51 (42.9)	68 (57.1)
Indeterminate strictures (PSC patients)	82	36 (43.9)	46 (56.1)
Cystic lesions of the pancreas (incl. IPMN)	64	22 (34.4)	42 (65.6)
Chronic pancreatitis ± lithotripsy	20	11 (55.0)	9 (45.0)
Miscellaneous	22	7 (31.8)	15 (68.2)

## Discussion

Our study represents the largest single institution experience in the use of the SOPCP (SpyGlass) technique in routine clinical practice. Our results suggest it offers important options for direct intraluminal visual inspection, with the added possibility of targeted biopsies and therapeutic intervention in both the biliary (71%), and the pancreatic (23%) ductal systems. SOPCP significantly impacted or solved the clinical problem in 64% of the cases. The currently used grading system for diagnostic and therapeutic yield of SOPCP carries a risk of bias towards grade 3–4 scoring, and this issue will have to be addressed in a future validation study to establish inter-observer reliability. A significant caveat accompanying the use of SOPCP, is the 16% risk of adverse events.

The Spyglass System was first devised and clinically tested in 2005–2006 by Chen and Pleskow [17]. Since then, various groups have reported their initial experience, although numbers are often small, data collection retrospective and outcome measures varied. Procedural success has consistently been reported by most investigators to be above 90% [19–26].

The most frequent indication for SOPCP in our patient population was the characterisation of indeterminate biliary strictures, one of the most challenging tasks clinicians are often faced with. Conventional methods of investigation such as cross-sectional imaging, as well as emerging modalities such as endoscopic ultrasonography and intraductal ultrasonography, are plagued with diagnostic uncertainty. Attempts at tissue sampling during ERCP with either brush cytology and/or ERCP-directed biopsy, have not yielded consistent results [27–31]. Two recent meta-analyses defined the sensitivity and specificity of SOPCP to diagnose indeterminate strictures [4, 32]. Both revealed a high sensitivity of visual impression (84.5–90%) for evaluating strictures as malignant, with a high specificity for SpyBite biopsy (98%) as confirmation. One of the limitations with analyses attempting to define diagnostic capability, is the possible lack of a ‘gold standard’ for verification of the diagnosis. Accordingly, with benign strictures of the bile duct, such as PSC, corroborating histology is usually unavailable. In 56–57% of our patients undergoing SOPCP for the evaluation of indeterminate bile duct strictures, the attributed clinical value was graded as high (grade 3–4). Our results are consistent with previously published data indicating the persistent diagnostic challenge regarding indeterminate strictures, but the final assessment of the diagnostic precision of the system, has to be further elucidated within the frame of large prospective series with adequate standards for comparison.

An important therapeutic indication for cholangioscopy is the treatment of ‘difficult’ bile duct stones, where duct clearance cannot be achieved by conventional means. A recent meta-analysis on the overall performance of all types of peroral cholangioscopy reported a stone clearance rate of 88% for bile duct stones [33]. Both electrohydraulic and holmium laser lithotripsy can be delivered via the SpyGlass system, and in our study complex cholelithiasis was the indication in 16% of cases. Although this is a small percentage of the total SOPCP cohort in out experience, in 79% of these procedures the therapeutic value of SOPCP was graded as high (grade 3–4). While our results draw attention to the established role of cholangioscopy in the treatment of complex biliary stone disease, it also suggests least therapeutic benefit for stones associated with chronic pancreatitis.

In our experience the most common indication for pancreatoscopy was a cystic lesion of the pancreas, including IPMN, and in 66% of these procedures, diagnostic yield was high (grade 3–4). The malignant potential of cystic lesions of the pancreas is notoriously difficult to accurately determine from conventional investigation methods, leading to complex decision making when choosing a treatment strategy [34, 35]. In patients with established or suspected IPMN we observed that at the time of pancreatoscopy, intraductal accumulation of mucous was a consistent finding.

There are certain technical limitations that warrant discussion. This study evaluates the first generation of the Spyglass system, where image quality has been one of the major drawbacks. The development of high-definition video technology will certainly enhance image quality [36]. Considering the current constraints, visualization can be optimized by free irrigation of saline, and adequate suctioning and drainage via a generous sphincterotomy.

Pancreatitis was the most frequent adverse event after SOPCP in our study, and like others [2, 20, 23, 37], we acknowledge this risk. It is not surprising that introduction of the device into, and manipulation inside of the main pancreatic duct emerged as a significant risk factor for post procedural pancreatitis. In our experience, irrigation with saline is necessary to clear the endoscopic view, and gentle handling and careful advancement of the device through the central parts of the main duct is mandatory to avoid damage. Although not allowing for further statistical analysis, a significant number of cases that developed pancreatitis had a main pancreatic duct with a normal diameter. Whether other procedure related factors play a role in the risk for pancreatitis is still unknown. One lethal adverse event is serious enough to highlight the need for careful dissemination of this technology outside of dedicated, specialized centers. Future studies will further our understanding of how adverse event risk can be minimized.

Our study was not a prospectively controlled analysis, but rather observational in design, assessing data from consecutive patients undergoing SOPCP. This can introduce bias toward visual impression, if the clinical history and results of prior investigations are known to the endoscopist. However, because we are scrutinising a new and evolving technical procedure, we believe that the endoscopist should have as much clinical information available as possible to maximize diagnostic and therapeutic yield at the time of the investigation.

## Conclusion

SOPCP offers unique information from intraluminal visual inspection and therapeutic intervention of the biliary (71%) and pancreatic ducts (23%), but is burdened by a 16% risk of adverse events. We found the procedure to have significant clinical value in 64% of cases, firmly establishing its role as an important diagnostic and therapeutic adjunct to ERCP, and mandating further careful and critical exploration.

## Abbreviations

AER: Adverse event rate
ASA: American Society of Anesthesiologists
ASGE: American Society for Gastrointestinal Endoscopy
ERCP: Endoscopic retrograde cholangiopancreatography
EUS: Endoscopic ultrasound
IPMN: Intraductal papillary mucinous neoplasms
PSC: Primary sclerosing cholangitis
SOPCP: Single-operator cholangiopancreatoscopy
